# Metabolic acidosis and the progression of chronic kidney disease

**DOI:** 10.1186/1471-2369-15-55

**Published:** 2014-04-03

**Authors:** Wei Chen, Matthew K Abramowitz

**Affiliations:** 1 Division of Nephrology, Department of Medicine, Albert Einstein College of Medicine, Bronx, NY, USA; 2 Department of Epidemiology & Population Health, Albert Einstein College of Medicine, 1300 Morris Park Avenue, Ullmann 615, Bronx, NY 10461, USA

**Keywords:** Bicarbonate, Dietary acid, Net endogenous acid production, Sodium bicarbonate, Alkali, Ammonia, Complement, Endothelin, Aldosterone

## Abstract

Metabolic acidosis is a common complication of chronic kidney disease. Accumulating evidence identifies acidosis not only as a consequence of, but as a contributor to, kidney disease progression. Several mechanistic pathways have been identified in this regard. The dietary acid load, even in the absence of overt acidosis, may have deleterious effects. Several small trials now suggest that the treatment of acidosis with oral alkali can slow the progression of kidney disease.

## Review

Metabolic acidosis is a common complication of chronic kidney disease (CKD). Based on a cross-sectional analysis of the National Health and Nutrition Examination Survey, an estimated 26 million adults in the United States have CKD, and approximately 700,000 individuals have an estimated glomerular filtration rate (eGFR) less than 30 mL/min/1.73 m^2^[1]. As 30-50% of those with eGFR <30 mL/min/1.73 m^2^ have metabolic acidosis [2-4], approximately 200,000 to 350,000 individuals with CKD stage 4 and 5 have chronic metabolic acidosis in the United States.

Chronic metabolic acidosis may have various adverse effects in patients with CKD, including altered skeletal metabolism [5], insulin resistance [6], protein-energy wasting [7-9], and accelerated progression of kidney disease. In epidemiologic studies, low serum bicarbonate levels have been associated with high mortality (Table 1). In a study of 1,240 male patients with non-dialysis dependent CKD, the lowest mortality was observed among those with baseline serum bicarbonate levels of 26–29 mEq/L, whereas patients with levels <22 mEq/L had a 43% higher risk of mortality [10]. Using data from the African American Study of Kidney Disease and Hypertension (AASK) trial, Raphael et al. showed that higher bicarbonate levels within the range of 20 to 30 mEq/L were associated with reduced risk of the clinical composite outcome of death, dialysis or worsening renal function [11]. This review focuses specifically on the effect of metabolic acidosis on the progression of kidney disease.

**Table 1:** Observational studies of serum bicarbonate and long-term outcomes in persons without end-stage renal disease

**Study**	**Population**	**Main outcome(s)**	**Findings**	**pH or pCO**_**2 **_**available**	**Strengths**	**Limitations**
Shah et al. 2009 [4]	5,422 outpatients in the Bronx, NY; 9% with eGFR < 60 mL/min/1.73 m^2^	Kidney disease progression, defined as 50% decrease in eGFR or eGFR < 15 mL/min/1.73 m^2^	HR 1.54 (95% CI 1.13-2.09) for progression, for serum bicarbonate ≤22 mEq/L compared with 25–26 mEq/L	No	• Ethnically diverse cohort	• Single measure of serum bicarbonate
						• Data derived from clinical and administrative dataset
Menon et al. 2010 [12]	1,781 participants (839 randomized, 942 non-randomized) from the MDRD study	(1) ESRD (need for dialysis or transplantation); (2) all-cause mortality; (3) composite of 1 and 2	HR 1.05 (0.87-1.28), 0.99 (0.75-1.13), 1.04 (0.87-1.24) for need for kidney failure, all-cause mortality, and composite outcome, respectively, for serum bicarbonate 11–20 compared with 26–40 mEq/L	No	• Well-characterized cohort	• Single measure of serum bicarbonate
					• Adjustment for measured GFR	
Raphael et al. 2011 [11]	1,090 participants of the AASK trial	Composite outcome of death, ESRD (dialysis or transplantation), or GFR event (defined as a GFR reduction by 50% or by 25 ml/min/1.73 m^2^ from baseline)	HR 0.960 (0.924-0.998) for composite outcome, per mEq/L higher baseline serum bicarbonate	No	• Well-characterized cohort	• Single measure of serum bicarbonate
					• Adjustment for measured GFR	
					• Adjustment for errors in measurement of GFR and proteinuria	
Kovesdy et al. 2009 [10]	1,240 adults at a Veterans Affairs Medical Center; 87% with CKD stages 3 and 4	(1) All-cause mortality; (2) composite of predialysis mortality and initiation of dialysis	U-shaped association; HR for mortality 1.43 (1.10-1.87) for serum bicarbonate <22 compared with 26–29 mEq/L; similar results for composite outcome	No	• Adjustment for time-varying serum bicarbonate levels	• Data derived from clinical and administrative dataset
Navaneethan et al. 2011 [13]	41,749 outpatients with eGFR < 60 mL/min/1.73 m^2^ in Cleveland, OH	All-cause mortality	U-shaped association; HR 1.23 (1.16-1.31) for bicarbonate <23 compared with 23–32 mEq/L; HR 1.59 (1.49-1.69) for reaching bicarbonate <23 mEq/L	No	• Examination of temporal change in serum bicarbonate	• Data derived from clinical and administrative dataset
					• Large sample size	
Dobre et al. 2013 [14]	3,939 participants from the CRIC study	(1) Renal outcome, defined as 50% decrease in eGFR or ESRD (dialysis or transplantation); (2) atherosclerotic events; (3) CHF events; (4) all-cause mortality	Per mEq/L higher serum bicarbonate, HR 0.97 (0.94-0.99) for renal outcome; 0.99 (0.95-1.03) for atherosclerotic event; 1.14 (1.03-1.26) for CHF for serum bicarbonate ≥24 mEq/L; 0.98 (0.95-1.02) for mortality	No	• Well-characterized cohort	• Single measure of serum bicarbonate
Kanda et al. 2013 [15]	113 Japanese patients ≥60 years old with eGFR < 60 mL/min/1.73 m^2^	Kidney disease progression, defined as 25% decrease in eGFR or initiation of dialysis	HR 0.791 (0.684-0.914) for progression, per mEq/L higher serum bicarbonate	No	• Focus on elderly cohort	• Single measure of serum bicarbonate
						• Small sample size
Raphael et al. 2013 [16]	15,836 participants of NHANES III	All-cause mortality	HR 1.75 (1.12-2.74), 1.56 (0.78-3.09), and 2.56 (1.49-4.38) for total population, non-CKD, and CKD subgroups, respectively, for serum bicarbonate <22 mEq/L compared with 26–30 mEq/L	No	• Nationally representative cohort	• Single measure of serum bicarbonate
					• Compared CKD and non-CKD subgroups	

*Abbreviations*: HR, hazard ratio; CI, confidence interval; GFR, glomerular filtration rate; MDRD, Modification of Diet in Renal Disease; AASK, African American Study of Kidney Disease and Hypertension; ESRD, end-stage renal disease; CKD, chronic kidney disease; CRIC, Chronic Renal Insufficiency Cohort; CHF, congestive heart failure; NHANES, National Health and Nutrition Examination Survey.

### Pathophysiology leading to acidosis in CKD

Under normal conditions, the extracellular H^+^ concentration is tightly regulated and varies little from the normal value of approximately 40 nanomol/L. Regulation of acid–base homeostasis involves three basic steps: chemical buffering by extracellular and intracellular buffers, alteration of alveolar ventilation, and changes in renal H^+^ excretion [17]. The kidneys regulate H^+^ excretion by reabsorbing filtered HCO_3_^−^ and generating new HCO_3_^−^ in response to various stimuli. Secreted H^+^ combine with urinary buffers such as HPO_4_^2−^ and ammonia. In CKD the reduction in functioning nephrons causes defects in renal excretion of acid, mainly in the form of ammonium [18]. A fraction of patients may have inappropriate bicarbonate wasting in the urine [19].

The acidosis in CKD usually remains relatively stable. In uncomplicated acidosis, the serum bicarbonate levels typically are >12 mEq/L and blood pH is >7.2 [19,20]. There are two general possibilities for this stabilization of serum bicarbonate levels. One is that after an initial period of acid retention, the excretion and production of acid equalize. The other possibility is that acidosis evokes extrarenal mechanisms that dispose of the endogenous acid not excreted in the urine. The major source of such buffering would be base derived from bone. Carefully conducted balance studies in CKD patients with acidosis found a positive acid balance of approximately 10–20 mEq per day and negative calcium balance that improved with correction of acidosis [21,22]. However, the degree of acid retention has been debated, with the suggestion that these findings resulted from systematic measurement error [23]. While the magnitude of acid retention may be less than reported, it seems likely that acid production does exceed excretion in the setting of chronic renal acidosis [24].

The acidosis in mild to moderate CKD is predominantly hyperchloremic, with an increased anion gap observed variably and generally only with advanced CKD [25-27]. However, accounting for changes in serum albumin and other electrolytes in the anion gap calculation reveals a slightly more nuanced view. This maneuver reveals minor elevations in the anion gap, which are associated with decrements in serum bicarbonate, even in the CKD stage 2 eGFR range [28]. Although small, these differences in anion gap are independently associated with mortality [28]. Thus, there may be low-level accumulation of organic solutes even when the GFR is relatively preserved that partially accounts for changes in the serum bicarbonate level.

### Metabolic acidosis and progression of kidney disease

Epidemiologic studies have demonstrated that lower serum bicarbonate is associated with an increased risk of kidney disease progression (Table 1). In a single center retrospective cohort study involving patients with and without kidney disease, the risk of CKD progression was 54% (95% confidence interval (CI) 13-109%) higher for patients with bicarbonate levels <22 mEq/L compared with bicarbonate levels of 25 to 26 mEq/L [4]. Among 1,781 participants with CKD stages 2–4 from the Modification of Diet in Renal Disease Study, compared with serum bicarbonate levels ≥26 mEq/L, levels ≤20 mEq/L were associated with a higher risk of kidney failure (hazard ratio (HR) 2.22 (95% CI, 1.83-2.68)) while adjusting for demographic and cardiovascular disease factors, serum albumin, proteinuria, and cause of kidney disease [12]. However, the association was substantially attenuated and non-significant after adjustment for GFR, measured using iothalamate clearance (HR 1.05 (95% CI, 0.87-1.28)). The implication was that lower serum bicarbonate was a marker for more advanced kidney disease, and that analyses adjusting for estimated GFR were limited by residual confounding. However, Raphael et al. performed a similar analysis using data from 1,094 African American participants of the AASK trial with GFR measured by iothalamate clearance [11]. After controlling for measured GFR, higher serum bicarbonate in the 20 to 30 mEq/L range was associated with a lower risk of dialysis or worsening kidney function (HR 0.932 (95% CI, 0.881-0.986) per 1 mEq/L serum bicarbonate). Most recently, in 3,939 participants with CKD stages 2–4 in the Chronic Renal Insufficiency Cohort, every 1 mEq/L higher serum bicarbonate level was associated with a 3% (95% CI, 1-6%) lower risk of developing end-stage renal disease or a 50% decline in eGFR during follow-up [14].

**Figure 1:**
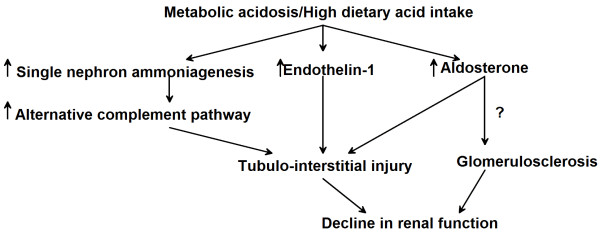
**Pathogenesis of kidney disease progression due to chronic metabolic acidosis.** Metabolic acidosis and/or a high dietary acid load may contribute to progressive kidney disease through multiple mechanisms, including increased ammonia generation per nephron leading to activation of the alternative complement pathway and increased endothelin-1 and aldosterone levels in the kidney. Each of these factors may cause tubule-interstitial injury leading to a decline in kidney function. Hyperaldosteronism could also accelerate glomerulosclerosis, although glomerular injury due to metabolic acidosis has not been reported in animal models.

Several factors have been implicated in the effect of metabolic acidosis on the progression of kidney disease. These include ammonia-induced complement activation and increased production of endothelin and aldosterone (Figure 1). Although total ammonium excretion decreases with progressive CKD, ammonia generation per nephron actually increases [29]. This adaptive response may be deleterious for the surviving nephrons [30]. Nath et al. examined the role of ammonia in the pathogenesis of tubulo-interstitial injury using the rat remnant kidney model [31]. Chronic sodium bicarbonate supplementation lowered renal vein total ammonia concentrations, reduced tubular deposition of complement components C3 and C5b-9, and ameliorated structural and functional evidence of tubulo-interstitial damage. The authors proposed that ammonia, as a nitrogen nucleophile, reacted biochemically with C3 to trigger the alternative complement pathway. Therefore, the compensatory increase in single-nephron ammoniagenesis observed in CKD could further instigate progressive kidney injury.

Acidosis has been shown to increase endothelin (ET)-mediated tubulo-interstitial injury as well. ET is an endothelial cell-derived peptide with 3 mammalian isoforms: ET-1, ET-2, and ET-3. The kidneys produce ET-1 in relatively high amounts and contain abundant ET receptors, particularly in the vasculature and the medulla [32]. *In vitro* evidence has demonstrated that ET-1 promotes the synthesis of fibronectin and collagen [33]. Several *in vivo* studies have correlated renal ET-1 gene expression and urinary excretion with the degree of proteinuria and glomerular and tubulo-interstitial damage [34]. ET regulates multiple renal functional parameters, one of which is acid–base handling. Specifically, ET-1 mediates increased renal acid excretion in response to a systemic acid challenge. Rats given a dietary acid load as (NH_4_)_2_SO_4_ increased ET-1 addition to renal interstitial fluid, and pharmacological inhibition of ET receptors using bosentan blunted distal tubule acidification [35]. Using a remnant kidney model, Wesson and colleagues demonstrated that dietary protein-induced tubulo-interstitial injury and GFR decline were mediated by ET, and that alkali supplementation prevented these effects [36,37].

Excess aldosterone could also mediate the decline in GFR caused by acidosis, via its hemodynamic effects and its pro-fibrotic actions [38]. Hyperaldosteronism in the setting of reduced nephron mass contributes to hypertension, proteinuria and glomerulosclerosis in the remnant kidney model [39,40]. Chronic aldosterone treatment plus a high salt diet produced hypertension and severe glomerular and tubulo-interstitial injury in non-nephrectomized rats [41,42]. Schambelan et al. examined the effect of chronic metabolic acidosis on adrenocortical hormone production by administering NH_4_Cl for 5 days to four normal subjects, and found that chronic metabolic acidosis induced a sustained stimulation of aldosterone secretion [43]. Aldosterone mediates increased distal nephron acidification in the setting of metabolic acidosis, and prevention of kidney disease with alkali in the rat remnant kidney model was associated with reduced aldosterone production in kidney cortex [44,45].

More recently, the effect of acidosis on kidney injury has been explored using gene expression analyses. Raj et al. examined the effect of exposure of Madin-Darby canine kidney cells (an immortalized cell line derived from the dog distal renal tubule) to medium with pH 7.4 and 7.0 for 24 hours, and found that acid stress upregulated the expression of genes that might participate in the pro-inflammatory process leading to glomerulosclerosis and fibrosis [46].

### Dietary acid, acid retention and progression of kidney disease

The net dietary acid load is determined by the balance of acid-forming and base-forming precursors in the diet. Common foods that provide a high dietary acid load include cheese, meat, eggs and grains, whereas fruits and vegetables are rich in alkali precursors [47,48]. Generally speaking, diets high in animal protein produce high levels of net endogenous acid production (NEAP), whereas vegan and vegetarian diets result in low, or even negative, NEAP. Low NEAP can be achieved even while preserving the proportion of energy derived from dietary protein. This was demonstrated in the Dietary Approaches to Stop Hypertension (DASH) trial, where a diet incorporating increased fruit and vegetable intake produced substantially lower NEAP than the typical American diet [47].

The usual diet consumed in the Western world is quite different from that which our ancestors ate. Ancestral human diets were largely plant-based, and were characterized by much lower levels of refined carbohydrates and sodium, and much higher levels of fiber and potassium, than contemporary diets [49,50]. Sebastian et al. estimated the net acid load from retrojected ancestral pre-agricultural diets and compared it with contemporary diets [51]. They found that 87% of 159 retrojected pre-agricultural diets were net base-producing with a mean NEAP of −88 mEq per day, compared with +48 mEq per day for the average American diet. Similarly, Strohle et al. examined the dietary net acid load for 229 worldwide historically-studied hunter-gatherer societies, and confirmed that the NEAP became progressively more positive as the dietary plant-to-animal ratio declined [52]. The historical shift from negative to positive NEAP, or from net base-producing to acid-producing, is thought due to the displacement of high-bicarbonate-yielding plant foods in the ancestral diet by cereal grains and energy-dense, nutrition-poor foods in the contemporary diet. It has been postulated that on an evolutionary scale this change has occurred rapidly, in a time span too brief for adequate genetic adaptation [53-55]. The resultant mismatch between the modern Western diet and a core metabolic machinery that was selected for 50,000 to 100,000 years ago may therefore contribute to chronic disease [56].

The high dietary acid load of contemporary diets could impair kidney function by inducing metabolic acidosis or even subclinical acid retention. In rats, acid loading increased blood and renal cortical acid content measured by microdialysis, consistent with net acid retention [57]. Despite no difference in blood pH or serum bicarbonate, rats with reduced nephron mass had higher tissue acid content compared with control animals. Dietary acid accelerated GFR decline, which was ameliorated by dietary alkali [58]. The decline in GFR caused by dietary acid could be mediated by increased kidney ET and aldosterone production [45,59] (Figure 1).

In humans, higher NEAP has been associated with lower serum bicarbonate in persons with kidney disease and in the general population [60,61]. Furthermore, this association is strongest among those with more advanced CKD and among middle-aged and older individuals, populations in whom the capacity to excrete an acid load is relatively impaired [60,61]. Among 632 participants from the AASK cohort study, higher NEAP was associated with a faster decline in GFR over a median follow up of 3.2 years [62]. Thus, animal studies and epidemiologic data link dietary acid load with the pathogenesis of progressive CKD.

### Treatment

Several single-center clinical studies have examined the effect of alkali therapy on the progression of kidney disease. In a 2-year, open-label, randomized trial, 134 patients with CKD stage 4 and serum bicarbonate levels of 16 to 20 mEq/L were assigned to receive oral sodium bicarbonate or to continue routine care [63]. Sodium bicarbonate was provided as 600 mg tablets dosed thrice daily and increased as necessary to maintain bicarbonate levels ≥23 mEq/L. Compared with the control group, bicarbonate-supplemented patients experienced a slower decline in creatinine clearance (5.93 vs. 1.88 mL/min/1.73 m^2^; p < 0.0001) and fewer developed ESRD. This was followed by another prospective interventional study of 59 patients with a clinical diagnosis of hypertensive nephropathy, all treated with regimens including an angiotensin-converting enzyme (ACE) inhibitor, who had an eGFR 20–59 mL/min/1.73 m^2^ and a serum bicarbonate <22 mEq/L [64]. Thirty patients prescribed sodium citrate were compared with the remaining 29 patients who were unable or unwilling to take sodium citrate or bicarbonate. After 24 months, patients taking sodium citrate had lesser eGFR decline and significantly lower urinary ET-1 excretion and tubulo-interstitial injury, as measured by urinary N-acetyl-β-D-glucosaminidase (NAG). In a second study by the same group, 120 patients with hypertensive nephropathy and CKD stage 2, all taking ACE inhibitors, were randomized in a blinded fashion to receive 0.5 mEq/kg/day sodium bicarbonate, sodium chloride, or matching placebo. After 5 years, the rate of eGFR decline was slowest in the patients randomized to sodium bicarbonate, and they had lower urine ET-1 and NAG compared with the other groups [65]. Of note, the mean serum bicarbonate at study entry among these 120 participants was 26.2 mEq/L, well within the normal range.

More recently, dietary modification has been examined by Goraya and colleagues [66,67]. In patients with CKD stage 2 due to hypertensive nephropathy, 30 days of increased fruit and vegetable consumption produced similar reductions in urinary NAG and albuminuria as did oral sodium bicarbonate [66]. Similar results were found in 71 patients with stage 4 CKD and serum bicarbonate <22 mEq/L who were randomized to 1 year of sodium bicarbonate at 1.0 mEq/kg per day or increased fruits and vegetables to reduce dietary acid by half [67]. The serum bicarbonate increased with the dietary intervention, although less than in the bicarbonate group, whose alkali dose would be expected to nearly completely neutralize the dietary acid load. The aforementioned markers of kidney injury declined similarly in the 2 groups. It is encouraging that there were no complications from hyperkalemia, but it should be noted that only patients with plasma K^+^ ≤4.6 mEq/L were enrolled in the study.

Thus, accumulating evidence suggests that treatment of chronic metabolic acidosis could slow the progression of CKD. However, the evidence base is not yet definitive. A systemic review was performed examining the published literature regarding alkali therapy through July 2011 [68]. The authors concluded that although oral alkali might afford a long-term benefit in slowing the progression of CKD, differences in study protocols and small sample sizes precluded definitive conclusions. Additional, more nuanced questions also remain unanswered. If alkali therapy does retard progression, what serum bicarbonate level should be targeted? Is there a risk to overcorrection of acidosis? Higher serum bicarbonate was associated with increased risk of congestive heart failure in the CRIC study [14], but to date interventional studies have not demonstrated significant metabolic alkalosis or fluid overload with even high-dose alkali. Is serum bicarbonate even the right target, given evidence of acid retention and deleterious effects within the normal range of serum bicarbonate? To what extent should dietary interventions to reduce the dietary acid load be employed in lieu of, or in addition to, oral alkali? There are 3 ongoing randomized controlled trials of alkali therapy in patients with CKD that have the potential to answer some of these questions and to impact clinical practice (NCT01640119 [69]; NCT01452412 [70]; EUDRACT Number 2012-001824-36 [71]). A summary of these trials is provided in Table 2. Several smaller studies are examining other effects of base, including changes in urinary TGF-β1 (NCT01574157), vascular endothelial function (NCT02031770), and its role in sickle cell disease (NCT01894594).

**Table 2:** Summary of on-going randomized clinical trials of alkali therapy in patients with chronic kidney disease

**Title**	**Correction of Metabolic Acidosis with Use of Bicarbonate in Chronic Renal Insufficiency (NCT01640119)**[69]	**Alkali Therapy in Chronic Kidney Disease (NCT01452412)**[70]^**^**^	**Oral Sodium Bicarbonate Supplementation in Patients with Chronic Metabolic Acidosis and Chronic Kidney Disease (EUDRACT Number 2012-001824-36)**[71]
Estimated primary completion date	12/2013	1/2015	Not available^#^
Anticipated sample size	728	150	200
CKD stage	Stage 3b & 4	Stage 3 & 4	Stage 3 & 4
Serum bicarbonate levels at randomization	≥18 mEq/L	20-26 mEq/L	<21 mEq/L
Study design	Randomized, open label	Randomized, placebo-controlled, double blind	Randomized, open label
Intervention	Bicarbonate administration to keep bicarbonate levels between 24–28 mEq/L	Sodium bicarbonate 0.4 mEq / kg ideal body weight per day	Sodium bicarbonate with target bicarbonate levels of 24 ± 1 mEq/L
Control	No intervention, partial correction if bicarbonate <18 mEq/L (up to 22 mEq/L)	Placebo	Rescue therapy of sodium bicarbonate with target bicarbonate level of 20 ± 1 mEq/L
Locations	Multiple centers in Italy	2 centers in the United States (Bronx, NY and Cleveland, OH)	Single center in Vienna, Austria
Follow up length	36 months	24 months	24 months
Primary outcome	Doubling of Cr	HOMA-IR, sit to stand to sit speed, DEXA of wrist, urinary NGAL & KIM-1	Means of eGFR, calculated using the 4-variable-MDRD Study equation
Secondary outcome measures	All-cause death, start of dialysis	Glucose disposal rate by euglycemic hyperinsulinemic clamp, hand-grip strength, serum calcium, phosphate, 1,25-dihydroxyvitamin D, PTH, Cr, cystatin C, urinary albumin/Cr ratio, urinary cystatin	Death, need for renal replacement therapy, change in markers of bone metabolism

*Abbreviations:* CKD, chronic kidney disease; Cr, creatinine; HOMA-IR, homeostasis model assessment-estimated insulin resistance; DEXA, dual energy X-ray absorptiometry; NGAL, urinary neutrophil gelatinase-associated lipocalin; KIM-1, kidney injury molecule-1; PTH, parathyroid hormone; eGFR, estimated glomerular filtrate rate; MDRD, modification of diet in renal disease; NY, New York; OH, Ohio.

^Our institution is one of the centers for the study (NCT01452412) and one of us (MKA) is a co-investigator. The information presented is updated from clinicaltrials.gov.

^#^Recruitment was planned to start in October 2013.

## Conclusion

Patients with CKD develop metabolic acidosis due to reduced kidney mass and defects in renal acid excretion. Chronic metabolic acidosis is a common complication of CKD and appears to contribute to the progression of kidney disease. High dietary acid intake has also been demonstrated to worsen kidney function by induction of metabolic acidosis and/or subclinical acid retention. The factors that have been implicated in this effect of acidosis on CKD progression include ammonia-induced activation of the alternative complement system, as well as increased endothelin and aldosterone production. Existing evidence from clinical trials suggests that alkali therapy could retard the progression of CKD. Increased fruit and vegetable consumption appears to be a reasonable alternative to alkali therapy for patients with mild metabolic acidosis and without hyperkalemia. However, definitive evidence is lacking for optimal evidence-based practice guidelines. Ongoing trials will hopefully facilitate more evidence-based treatment of metabolic acidosis in the future.

## Competing interests

The authors declare that they have no competing interest.

## Authors’ contributions

WC drafted the manuscript and both WC and MKA revised it and approved the final manuscript. Both authors have read and approved the final manuscript.

## Pre-publication history

The pre-publication history for this paper can be accessed here:

http://www.biomedcentral.com/1471-2369/15/55/prepub
